# Supplementary material for: A framework for assessing the impact of accelerated approval
Source: PLoS One. 2022 Jun 24;17(6):e0265712. doi: 10.1371/journal.pone.0265712 (PMC9231718; doi:10.1371/journal.pone.0265712)
Supplement: S1 Appendix — (DOCX) [file pone.0265712.s001.docx]

**Appendix 1: Accelerated Approval**

Accelerated Approval is one of four programs identified in the current FDA guideline aimed at facilitating and expediting the development and review of new drugs to address unmet medical needs for treating serious or life threatening conditions.[[1](#_ENREF_1)] The following table presents an abbreviated summary of the four programs.

|  | | Fast Track | Breakthrough Therapy | Accelerated Approval | Priority Review |
| --- | --- | --- | --- | --- | --- |
| Nature of program | | Designation | Designation | Approval Pathway | Designation |
| Qualifying criteria | | • Intended to treat a serious condition AND nonclinical or clinical data demonstrate the potential to address unmet medical need OR • designated as a qualified infectious disease product | • Intended to treat a serious condition AND preliminary clinical evidence indicates that the drug may demonstrate substantial improvement on a clinically significant endpoint(s) over available therapies | • Treats a serious condition AND generally provides a meaningful advantage over available therapies AND demonstrates an effect on a surrogate endpoint that is reasonably likely to predict clinical benefit or on a clinical endpoint that can be measured earlier than irreversible morbidity or mortality (IMM) that is reasonably likely to predict an effect on IMM or other clinical benefit | • An application (original or efficacy supplement) for a drug that treats a serious condition AND, if approved, would provide a significant improvement in safety or effectiveness OR • Any supplement that proposes a labeling change pursuant to a report on a pediatric study under 505A OR  • An application for a drug that has been designated as a qualified infectious disease product |
| Features | | • Actions to expedite development and review  • Rolling review | • Intensive guidance on efficient drug development  • Organizational commitment  • Rolling review | • Approval based on an effect on a surrogate intermediate clinical endpoint that is reasonably likely to predict a drug’s clinical benefit | • Shorter clock for review of marketing application (6 months compared with the 10-month standard review) |
| Additional considerations | • May be rescinded if it no longer meets the qualifying criteria | • May be rescinded if it no longer meets the qualifying criteria | • Confirmatory trials to verify and describe the anticipated effect on IMM or other clinical benefit  • Subject to expedited withdrawal | • Designation will be assigned at the time of original BLA, NDA, or efficacy supplement filing |  |

The Accelerated Approval program applies to drugs that treat serious conditions and fulfill an unmet medical need based on a surrogate endpoint. A surrogate endpoint is a marker, such as a laboratory measurement, radiographic image, physical sign or other measure that is thought to predict clinical benefit, but is not itself a measure of clinical benefit. An application for accelerated approval should also include evidence that a proposed surrogate endpoint or an intermediate clinical endpoint is reasonably likely to predict the intended clinical benefit of a drug. The evidence that provides support for the use of a surrogate endpoint will depend in the circumstances but usually will be based on considerations such as whether they measure the underlying cause of the disease, an effect that predicts the ultimate outcome, or the state of the pathophysiologic pathway leading to the clinical outcome. Epidemiologic evidence can be useful here, but requires an assessment of whether there is reliable and consistent epidemiologic evidence supporting the relationship between the endpoint and the intended clinical benefit, how precisely the epidemiologic relationship between the endpoint and clinical outcome is defined, and whether the effect on the surrogate endpoint has been shown to predict a clinical benefit with another drug or drugs.

A serious condition is a disease or condition associated with morbidity that has substantial impact on day-to-day functioning that clinical judgment suggests will progress in severity or lead to mortality if left untreated. The drug in question must be intended to have an effect directly or indirectly on a serious condition, including mitigating or preventing a serious treatment-related side effect or avoiding or diminishing a serious AE associated with available therapy.

An unnmet medical need is a condition whose treatment or diagnosis is not addressed adequately by available therapy. If there is no available therapy for a serious condition, there is clearly an unmet medical need. A new treatment could be considered to address an unmet medical need even when available therapy exists, under certain conditions. [[1](#_ENREF_1)]

The guidance states that FDA may grant accelerated approval to a product for a serious or life-threatening disease or condition upon a determination that the product has an effect on a surrogate endpoint that is reasonably likely to predict clinical benefit, or on an intermediate clinical outcome ascertainable sooner than irreversible morbidity or mortality that is reasonably likely to predict an effect on irreversible morbidity or mortality. The accelerated approval pathway has been used primarily in settings in which the disease course is long and an extended period of time would be required to measure the intended clinical benefit of a drug. Accelerated approval also may be useful in acute disease settings where the clinical event for which benefit would be realized occurs rarely so that very large trials would be needed to demonstrate benefit.

The 2016 21^st^ Century Cures Act that mandated the establishment of programs for expedited approval of these drugs also required that that the FDA develop ‘patient-focused drug development guidance’ that addresses how the FDA plans to use patient experience data ‘with respect to the structured risk-benefit assessment framework’ described in the Federal Food, Drug, and Cosmetic Act, while adhering to the (substantial) evidence standard required by the FFDC Act. [[40](#_ENREF_40)] The principal risk of the accelerated approval approach is the possibility that patients will be exposed to a drug that ultimately will not be shown to provide an actual clinical benefit. Also, there generally will be fewer, smaller, or shorter clinical trials than is typical for a drug receiving traditional approval, which usually will mean there is less information about the occurrence of rare or delayed adverse events that subsequently will emerge and be incorporated into the label.[[35](#_ENREF_35)]

Consequently, drug companies still are required to conduct studies to confirm the anticipated clinical benefit with due diligence and promptly. In general, the confirmatory trial would evaluate a clinical endpoint that directly measures clinical benefit, ordinarily in the same disease population that was studied to support accelerated approval. If these confirmatory trials show that the drug actually provides a clinical benefit, then the FDA grants traditional approval for the drug. If they do not show that the drug provides clinical benefit, FDA has (in principle) regulatory procedures in place that could lead to removing the drug from the market.

The Accelerated Approval process has been in place for a number of years. A recent study evaluated the preapproval and confirmatory trials of drugs granted accelerated approval between 2009 and 2013. [[41](#_ENREF_41)] Accelerated approval was granted to 22 drugs for 24 indications (19 for treating cancer), based on a total of 30 preapproval studies. Eight of the studies included fewer than 100 participants, and 2/3 (20) of the studies included fewer than 200 participants. After 3 or more years of followup, half (19 of 38) of the required confirmatory studies were completed. Most importantly, clinical benefit had not been confirmed for 8 indications approved at least 5 years previously.
