# Supplementary material for: A framework for assessing the impact of accelerated approval
Source: PLoS One. 2022 Jun 24;17(6):e0265712. doi: 10.1371/journal.pone.0265712 (PMC9231718; doi:10.1371/journal.pone.0265712)
Supplement: S2 Appendix — (DOCX) [file pone.0265712.s002.docx]

**Appendix 2: Elements of a Statistical Decision Framework**

1. Data consisting of a variable X taking values in X = {x_1_, x_2_, ...} or some interval on the real line, etc. with a likelihood f_X_(x; θ) depending on parameter(s) θ ≡ “State of Nature”.
2. A prior distribution g(θ;ψ), θ ∈ Θ, for the unknown true value of θ; ψ = parameter(s) of the prior distribution of θ.
3. A decision procedure D(x) that identifies an action to be taken on observing X = x; D(x) is a member of a set D = {D_1_, D_2_, ...} of possible decision procedures. This is key because the point of obtaining the data is to provide guidance for choosing an appropriate action.
4. A set of possible actions A = {a_1_, a_2_, ... } consisting of the possible values of D(x). Different decision rules could specify different actions even with the same data.
5. A rule V(D(x), θ) that assigns a value to the action (in A) specified by D(x) when the true value of the State of Nature is θ. This rule has various names, e.g., loss function, utility function, etc. Assume for convenience that the rule is a loss function, so that small values are desirable. This relates a value to an action that the decision function D(x) specifies when x is given.
6. A risk function R(D,θ) corresponding to D that is the expected value of V with respect to x given θ,

$$R\left( D, \right)= \int V\left( D\left( x \right), \right)f_{X}\left( x; \right)\mathrm{dx}$$

(this would be a summation if the values in X are discrete). Every decision procedure D(x) has its own range of risks corresponding to the various “States of Nature” (values of θ). The risk value for any decision procedure D(x) reaches a maximum for some θ ∈ Θ. This depends on the random process that generates x values (i.e., the value of θ), but not on any particular value of x.

1. A minimax (or admissible) decision procedure D* is the decision procedure in D that has the smallest value of the maximum risk over the set of values of θ ∈ Θ; more formally,

D* = min_D∈D_ max_θ∈Θ_ R(D, θ)

1. The Bayes risk r_g_(D; ψ) of a decision procedure D is the expected value of the risk function R(D, θ) with respect to the prior distribution of θ,

r_g_(D; ψ) = $\int R(D,)g(;)d$

1. A Bayes Rule is the decision rule D^#^ in D that that provides the smallest value of r_g_(D; ψ) so that

r_g_(D^#^; ψ) = min_D∈D_ r_g_(D; ψ)

Every admissible procedure is a Bayes Rule.
